# Hydropersulfides (RSSH) Outperform Post-Conditioning and Other Reactive Sulfur Species in Limiting Ischemia–Reperfusion Injury in the Isolated Mouse Heart

**DOI:** 10.3390/antiox11051010

**Published:** 2022-05-20

**Authors:** Blaze M. Pharoah, Vinayak S. Khodade, Alexander Eremiev, Eric Bao, Ting Liu, Brian O’Rourke, Nazareno Paolocci, John P. Toscano

**Affiliations:** 1 Department of Chemistry, Johns Hopkins University, Baltimore, MD 21218, USA; bpharoa1@jhu.edu (B.M.P.); vkhodad1@jhu.edu (V.S.K.); aeremie1@jhu.edu (A.E.); ebao1@jhu.edu (E.B.); 2 Division of Cardiology, Johns Hopkins University School of Medicine, Baltimore, MD 21205, USA; tliu11@jhmi.edu (T.L.); bor@jhmi.edu (B.O.); 3 Department of Biomedical Sciences, University of Padova, 35131 Padova, Italy

**Keywords:** reactive sulfur species, hydrogen sulfide, hydropersulfides, carbonyl sulfide, cardioprotection, ischemia–reperfusion injury, post-conditioning, Langendorff, hypoxia-reoxygenation

## Abstract

Hydrogen sulfide (H_2_S) exhibits protective effects in cardiovascular disease such as myocardial ischemia/reperfusion (I/R) injury, cardiac hypertrophy, and atherosclerosis. Despite these findings, its mechanism of action remains elusive. Recent studies suggest that H_2_S can modulate protein activity through redox-based post-translational modifications of protein cysteine residues forming hydropersulfides (RSSH). Furthermore, emerging evidence indicates that reactive sulfur species, including RSSH and polysulfides, exhibit cardioprotective action. However, it is not clear yet whether there are any pharmacological differences in the use of H_2_S vs. RSSH and/or polysulfides. This study aims to examine the differing cardioprotective effects of distinct reactive sulfur species (RSS) such as H_2_S, RSSH, and dialkyl trisulfides (RSSSR) compared with canonical ischemic post-conditioning in the context of a Langendorff ex-vivo myocardial I/R injury model. For the first time, a side-by-side study has revealed that exogenous RSSH donation is a superior approach to maintain post-ischemic function and limit infarct size when compared with other RSS and mechanical post-conditioning. Our results also suggest that RSSH preserves mitochondrial respiration in H9c2 cardiomyocytes exposed to hypoxia-reoxygenation via inhibition of oxidative phosphorylation while preserving cell viability.

## 1. Introduction

Myocardial infarction (MI) is a leading cause of death and disability in the developed world and a significant socioeconomic burden [1]. In many cases, MI stems from the erosion or rupture of a vulnerable atherosclerotic plaque within a coronary artery, leading to a thrombus formation and subsequent occlusion of the same vessel. Prompt restoration of blood flow to the ischemic myocardium via a percutaneous procedure and fibrinolytic therapy is mandatory to limit infarct size. However, these interventions come with the cost of the so-called “reperfusion injury”, i.e., tissue damage caused by blood flow return to the myocardium [2]. Factors accounting for ischemia–reperfusion (I/R) injury have been studied for decades now, and yet there are no current pharmacological tools approved for its limitation in MI patients. 

Hydrogen sulfide (H_2_S) is an endogenously produced signaling molecule that regulates a myriad of physiological functions, including neurotransmission, vasodilation, and insulin secretion [3,4,5]. Several studies have demonstrated its ability to protect the myocardium against I/R injury in recent years [6,7,8,9,10,11]. For instance, the Lefer group was one of the first research teams to provide insights into the cardioprotective potential of H_2_S, using several animal models [12]. In addition to cardiac protection, Goodchild and colleagues reported that the daily administration of the H_2_S prodrug, SG-1002, preserves vessel number and density in ischemic limbs by increasing circulating H_2_S and nitric oxide (NO) levels [13].

Recently, H_2_S-related species, such as hydropersulfides (RSSH), polysulfides (RSS_(n)_SR, n ≥ 1), and inorganic polysulfide (HSS_(n)_H, n ≥ 1) have been found to account for many of the protective effects originally assigned to H_2_S [14,15]. Indeed, it is now increasingly manifest that a wide range of reactive sulfur species (RSS) can be enzymatically produced [16]. Recent progress in analytical methods has revealed that RSSH, (e.g., cysteine and glutathione hydropersulfides) and polysulfides are ubiquitous in mammalian and other biological systems [17]. Furthermore, Kevil and co-workers have demonstrated that RSS levels are markedly reduced in subjects with cardiovascular disease (CVD), suggesting RSS play a central role in maintaining the cellular redox homeostasis and, thus, cardiovascular health [18,19]. In the same vein, diallyl trisulfide has been shown to limit myocardial injury in a murine model of I/R injury [20]. Thus, RSS species may have bioregulatory roles similar to those ascribed to H_2_S, and their in situ coexistence complicates discerning which particular sulfur species is responsible for a given biological/pharmacological action. To address this conundrum, herein we compare the cardioprotective effects of H_2_S, RSSH, and a dialkyl trisulfide, each individually in the ex vivo Langendorff model. This approach permits testing whether these species have direct protective action, comparing their effectiveness on the ischemic heart independently from additional possible benefits such as those emanating from a better preserved systemic vascular function. By real-time monitoring heart contractility with this model, the preserved performance of the myocardium and tissue salvaging by bolus H_2_S administration is compared directly with that of slow H_2_S donation or sulfane sulfur supplementation via an RSSH donor or a dialkyl trisulfide on the post-ischemic heart. Because H_2_S has been reported to affect the metabolism of various tissue types in different disease models [21,22,23], ultimately offering protective properties, we also investigate the metabolic modulating capabilities of RSSH, which shows the highest cardiac cell-protective effects of the RSS studied herein. 

## 2. Materials and Methods

### 2.1. Reagents

Sodium sulfide (Na_2_S) was purchased from TCI chemicals. Cell Counting Kit-8 (CCK-8) was purchased from Dojindo Molecular Technologies, Inc. All other chemicals were purchased from Sigma Aldrich. H_2_S donor **1** and RSSH donors **2** and **3** were synthesized following reported procedures [24]. *N*-acetyl-*O*-ethyl cysteine trisulfide (**4**) was synthesized as shown in Scheme S1 (Supplementary Materials) [25,26]. Na_2_S (10 mM) stock solution was freshly prepared by dissolving it in molecular biology grade water (Corning). Stock solution of RSSH donors **2** and **3**, and sulfane sulfur donor trisulfide **4** were prepared in DMSO:Water (<0.001%) and diluted fresh each day before administration.

### 2.2. Animals

Male C57BL/6J mice obtained from Jackson Laboratories (Bar Harbor, ME, USA) were used for all experiments. Mice were between 12 and 14 weeks of age at the time of experimentation. All animals received humane care in compliance with the “Principles of Laboratory Animal Care” formulated by the National Society for Medical Research and the “Guide for the Care and Use of Laboratory Animals” published by the US National Institutes of Health. The Animal Care and Use Committee from Johns Hopkins University approved this study. 

### 2.3. Perfusion Experimental Protocol

**Figure 1:**
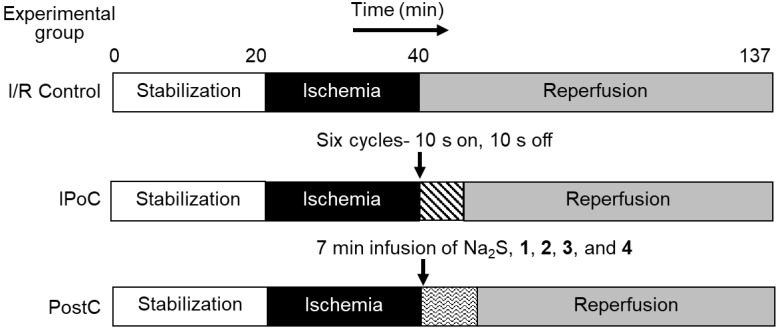
Schematic presentation of the Langendorff experimental protocol. Experimental groups: (1) I/R control, after equilibrium perfusion for 20 min, mouse hearts were subjected to 20 min of no-flow ischemia followed by 90 min reperfusion; (2) IPoC, after 20 min stabilization, mouse hearts were subjected to 20 min of no-flow global ischemia followed by alternating six cycles of 10-s intervals of reperfusion and ischemia and then reperfusion for 90 min; (3) PostC, after 20 min stabilization, mouse hearts were subjected to 20 min of no-flow global ischemia followed by infusion of 100 μM of Na_2_S, donors **1**, **2**, **3** and **4** independently for 7 min and then reperfusion for 90 min. Abbreviations: IPoC: ischemic post-conditioning, PostC: pharmacological post-conditioning.

As previously described [27,28], after anticoagulation with heparin and cervical dislocation, a thoracotomy was performed, and the heart was quickly excised and placed in ice-cold Krebs–Henseleit buffer (in mmol/L: 11.1 d-glucose, 1.2 MgSO_4_, 1.2 KH_2_PO_4_, 4.7 KCl, 118 NaCl, 2 CaCl_2_, 25 NaHCO_3_). The aorta was cannulated, and the heart was perfused with Krebs–Henseleit buffer (oxygenated with 95% O_2_/5% CO_2_ and maintained at pH 7.4) in a retrograde fashion at a constant pressure of 80 mmHg at 37 °C [29]. A catheter-linked latex balloon is inserted into the left ventricle bypassing the pulmonary veins. Changes in pressure were monitored, recorded, and processed using BIOPAC (Goleta, CA, USA) technology. After baseline equilibration for 20 min, we monitored heart rate-pressure product (RPP) and left-ventricular developed pressure (LVDP) (Table 1). Only those hearts displaying RPPs of 22,000 (or above) and LVDP of 60 mm Hg (or above) were included in the protocols to avoid poorly performing control hearts (either due to poor handling by the operator or issues intrinsic to the heart itself) confounding the final functional outcome. After that, the mouse heart was subjected to 20 min of no-flow global ischemia and 90 min of reperfusion. Mechanical post-conditioning was achieved by alternating six cycles of 10-s intervals of reperfusion and ischemia (20 s per cycle), as previously described [27]. Pharmacological postconditioning was performed by infusion of aqueous Na_2_S or compounds **1**, **2**, **3** or **4** (100 μM final concentration) at the onset of reperfusion for 7 min (Figure 1). Hearts were then reperfused for a total of 90 min. Control hearts received no pharmacological treatment or mechanical postconditioning. Coronary effluent was collected at the endpoint of reperfusion for control and RSSH-donor-**3**-treated hearts (1 mL).

### 2.4. Determination of Infarct Size

After the end of the protocols reported above, all hearts were dismounted from the rig. The aortic cannula was placed on an infusion line connected to a syringe pump (Harvard Apparatus). The mouse hearts were perfused with 1% (*w*/*v*) of 2,3,5-triphenyl tetrazolium chloride (TTC) [29] while sitting in a bath of 1% TTC at 37 °C for 10 min (Flow rate = 0.5 mL/min). Then, the pump is turned off and the heart remained in the TTC bath for an additional 5 min. Following incubation, the heart was de-cannulated, weighed, and cooled to a semi-frozen state at −20 °C for 20–30 min before the heart was sliced transversely at 1 mm thick slices. The slices were then weighed and fixed overnight in 10% formalin, followed by imaging with a high-resolution camera. Planimetry of the images was conducted using ImageJ (NIH). 

### 2.5. Estimation of Cardiac Injury Markers

Necrotic cell death was evaluated by analyzing the leakage of cardiac troponin I (cTnI) in the coronary effluent using commercially available ELISA methods [30]. 

### 2.6. Cell Culture

H9c2(2-1) embryonic rat heart myoblasts were obtained from the American Type Culture Collection. Cells were grown in Dulbecco’s minimal essential medium (DMEM), supplemented with fetal bovine serum (FBS) 10%. They were propagated in T75-flasks, split before reaching 70–80% confluence (usually every day or every second day), and used within 15 passages. Cells were passaged to tissue culture 96-well microtiter plates at the specified density in 200 μL volumes and incubated for 24 h. 

### 2.7. Hypoxia-Reoxygenation

Hypoxia/reoxygenation treatment was achieved by using a hypoxic chamber (New Brunswick, United States) to create a challenge of an in vitro hypoxia environment (1% O_2_) [31]. In brief, cells were firstly cultured in rich DMEM under normoxic conditions (95% air/5% CO_2_) at 37 °C for 24 h. Then, the rich media was removed, and nutrient deficient DMEM (without glucose, pyruvate or serum) that had been preequilibrated in the hypoxic chamber overnight was added (200 μL). The cells were placed in the chamber and incubated for the indicated time period. At the conclusion of the hypoxic episode, the media was replaced with rich DMEM with or without RSSH precursor **3** and then moved into a normoxic incubator for the indicated time. 

### 2.8. Viability

Undifferentiated H9c2 cells were seeded at a density of 5 × 10^3^ cells/well. After 24 h, the nutrient-rich DMEM was replaced with glucose, pyruvate, and serum-free DMEM (200 μL), and the cells were placed in a hypoxia chamber. Cells were incubated for 24 h under hypoxia before replacing media with nutrient-rich DMEM containing RSSH precursor **3** or vehicle and incubated under normoxic conditions for 3 h. At the end of of the “reperfusion” stage, each well was carefully washed three times with PBS (pH 7.4) before adding 100 μL of media (without-FBS) containing 10% *v*/*v* CCK-8 [32] and incubated for 3 h (95% air/5% CO_2_) at 37 °C prior to obtaining absorbance values at 450 nm. The relative % cell viability was calculated as 100 times the Abs450 (Hypoxia) ratio over Abs_450_ (Normoxia) for each condition tested. 

### 2.9. Oxygen Consumption Rate (OCR) via Seahorse Experiments

Mitochondrial respiratory function was monitored by changes in oxygen consumption rate (OCR). Briefly, H9c2 cells were plated at a density of 2 × 10^3^ cells per well into 96-well plates and incubated for 24 h. After 24 h, the nutrient-rich DMEM was replaced with glucose, pyruvate, and serum-free DMEM (200 μL) and the cells placed in a hypoxia chamber. Cells were incubated for 7 h under hypoxia before replacing media with nutrient-rich DMEM containing RSSH precursor **3** or vehicle, and incubated under normoxia for 3 h. At the completion of this “reperfusion” stage, each well was carefully washed three times with PBS (pH 7.4) before DMEM was replaced by Agilent Seahorse XF Base Medium containing 1 mM of pyruvate, 2 mM of glutamine, and 10 mM of glucose (adjusted pH to 7.4 with 0.1 N NaOH). The respiration modulating compounds were then loaded into the appropriate ports of a hydrated sensor cartridge and added sequentially following basal conditions. Cellular oxygen consumption rate (OCR), extracellular acidification rate (ECAR), and different indices were determined using the Seahorse XF96 analyzer (Agilent Technologies, Santa Clara, CA, USA). The final concentrations of compounds in the Seahorse XFp Cell Mito Stress Test were 1.5 μM oligomycin (a complex V inhibitor linked to ATP production), 2 μM FCCP (an uncoupling agent linked to maximal OCR), and 2 μM rotenone and antimycin A (complex I and III inhibitors, respectively, linked to non-mitochondrial OCR). Cell counting was used for normalizing Seahorse XF96 metabolic data to cellular number. Briefly, H9c2 cells in each well were fixed in 4% formalin for 10 min before nuclear staining with Hoescht 33342 (1:1000) in PBS. Cell nuclei were visualized on a Thermo Fisher EVOS fluorescence microscope and analyzed using ImageJ (NIH). The normalization unit of the present study was 1 × 10^3^ cells. Data were analyzed using Seahorse XF Cell Test Report data analysis. 

### 2.10. Statistical Analysis

Intergroup comparisons were performed by using a one-way analysis of the variance, followed by a post hoc Dunnett test with a family-wise alpha threshold of *p* < 0.05 (95% confidence interval) using GraphPad Prism software (Version 9.3.1). Analyzed data are represented as mean with standard error (mean ± SEM).

## 3. Results 

### 3.1. Reactive Sulfur Species/Donors Used in This Study 

**Scheme 1:**
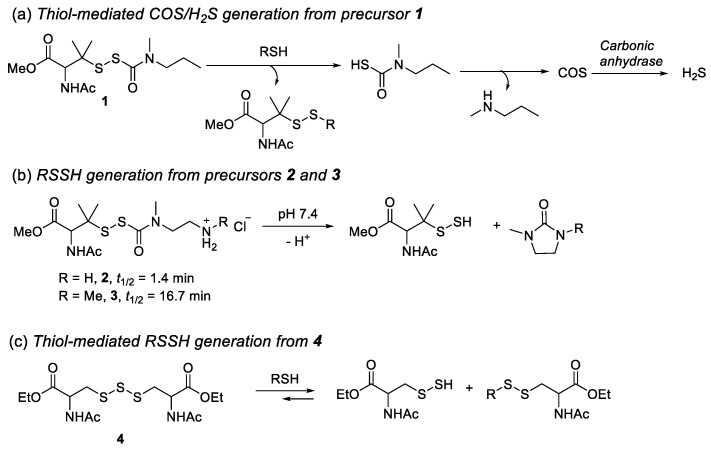
RSS donors used in this study.

The inorganic salt, sodium sulfide (Na_2_S), was used as the source of H_2_S. Because Na_2_S dissociates instantaneously in aqueous solution to produce high local H_2_S concentrations [33], which is different from the slow enzymatic generation in biological systems, we also utilized precursor **1** that produces H_2_S slowly. In the presence of biological thiols, **1** initially releases carbonyl sulfide (COS) (with a half-life of approximately 10 min under our experimental conditions) [24], which is rapidly hydrolyzed to H_2_S by carbonic anhydrase (Scheme 1a) [34]. Because of the intrinsically unstable nature, RSSH study is difficult and dependent on the use of donor molecules. In recent years, some small-molecule donors have been developed that efficiently release RSSH in response to various stimuli [35,36]. We utilized our recently developed RSSH precursors **2** and **3** (Scheme 1b) [24]. At pH 7.4 and at 37 °C, precursor **2** rapidly releases RSSH (t_1/2_ = 1.7 min), whereas **3** releases RSSH more slowly (t_1/2_ = 16.7 min). Cysteine trisulfide has been proposed as a reservoir of sulfane sulfur in the biological systems and has also been used to increase intracellular RSSH levels [37]. However, cysteine trisulfide is unstable under physiological conditions, likely stemming from deprotonation of the ammonium group at neutral pH and degradation via amine reactivity [38]. Hence, we have used *N*-acetyl-*O*-ethyl cysteine trisulfide (**4**) because of its stability and potentially enhanced bioavailability. We synthesized trisulfide **4** (Scheme S1) with high purity, relatively free of polysulfides. 

### 3.2. RSSH Improves the Rescue of Myocardial Function after I/R Injury over Na_2_S and Other H_2_S-Related Species

**Figure 2:**
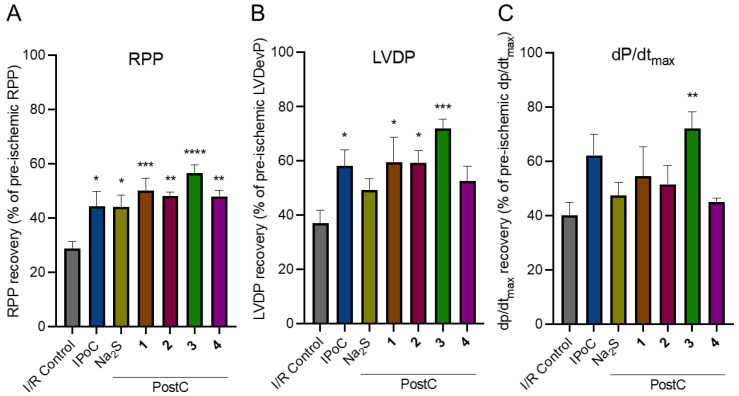
Cardioprotective effects induced by mechanical or pharmacological post-conditioning with different RSS at 100 μM. (**A**) Post-ischemic left ventricular RPP functional recovery; (**B**) Post-ischemic left ventricular developed pressure recovery; (**C**) Post-ischemic left ventricular rate of contraction. Results are expressed as the mean ± SEM using values obtained at 90 min of reperfusion normalized to the respective pre-ischemic value (n = 8 in each group) * *p*, 0.05, ** *p*, 0.005, *** *p*, 0.001, **** *p* < 0.0001 vs. I/R-Control.

We compared the efficacy of various RSS in preventing I/R injury with the protection afforded by classical post-conditioning. To this end, we used a global ischemia approach in Langendorff-perfused hearts and Na_2_S along with four different RSS donors (**1**, **2**, **3**, and **4**, as shown above). As expected, six cycles of 10-s episodes of ischemia and reperfusion at the onset of reperfusion (IpoC) increased the recovery of developed pressure from 35.0 ± 5.7 to 58 ± 6.7% as compared to control hearts receiving complete reflow at the onset of reperfusion (Figure 2B). Similarly, the percent recovery of the maximal rate of LV pressure rise (dP/dt_max_) also increased from 30 ± 4.3% to 63.3 ± 9.0% in the IPoC group. Consistent with previous reports [12,39,40], Na_2_S significantly improved the post-ischemic functional recovery. Cardiac RPP was 44.2 ± 11.1% in Na_2_S compared with 25.2 ± 8.6% in control I/R hearts (Figure 2A). Percent recoveries of LVDP, and dP/dt_max_ also increased following Na_2_S infusion (Figure 2B,C). Hearts treated with **1** displayed modest improvement in dP/dt_max_ after ischemia due to the benefits of slow and sustained release of H_2_S compared to bolus administration of Na_2_S. The fast release of RSSH from donor **2** exhibited similar protective properties to Na_2_S as did trisulfide **4**. However, RSSH donor **3** showed superior cardioprotection compared to all other RSS tested. RPP was 55.1 ± 7.6% in **3**-conditioned hearts vs. 25.2 ± 8.6% in control hearts. We report no significant difference in the values obtained at the end of stabilization for all groups tested as shown in Table 1. However, significant preservation of dp/dt_max_ is observed with RSSH precursor **3**, but with no notable impact on heart rate (Table 2). Consistently, we observed a two-fold increase over control in the recovery of LVDP and dP/dt_max_, confirming that RSSH donated by **3** positively impacts post-ischemic heart function in a manner that appears to be superior to all conditions tested.

### 3.3. RSS Limits Infarct Size after Global Ischemia/Reperfusion in Isolated Mouse Hearts

**Figure 3:**
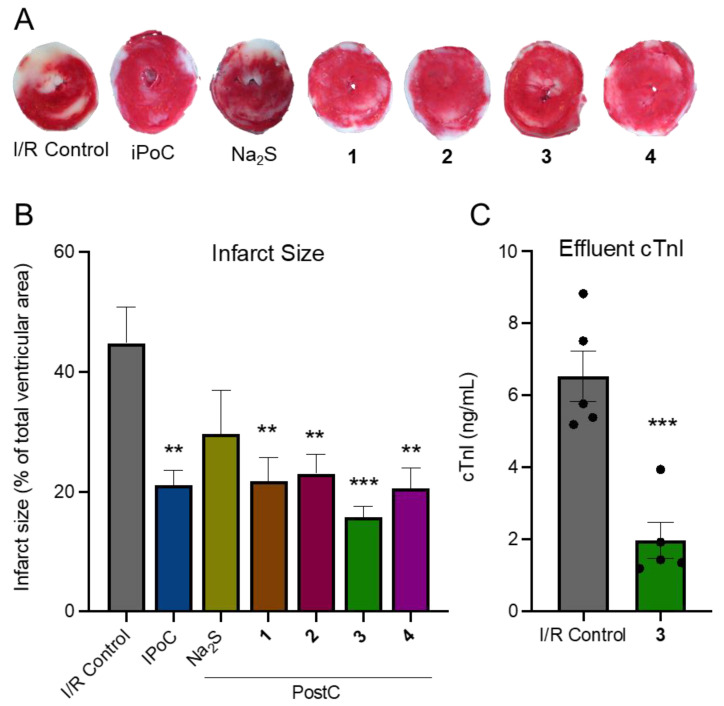
RSS reduce myocardial infarct size and pathological damage in isolated mice hearts subjected to I/R injury. (**A**) Representative images of TTC-stained transverse cardiac slices. (**B**) Quantitative graph of myocardial infarct size. The data represent the mean ± SEM (n = 8). (**C**) RSSH donor **3** reduces troponin-I level during reperfusion injury (n = 5). ** *p*, 0.005, *** *p*, 0.001 vs. I/R-Control.

To determine the extent to which the different RSS tested here prevent irreversible myocyte loss after I/R injury, we assessed the infarct size by TTC staining and planimetry. Active mitochondrial dehydrogenases convert the water-soluble compound TTC into an insoluble red precipitate, and the extent of staining correlates with the viable myocardium [41]. TTC staining demonstrates that 20 min of global ischemia followed by 90 min of reperfusion resulted in 45% infarcted volume of the heart (Figure 3A,B). Post-conditioning reduced infarct size by more than 20% with respect to the I/R control group. Na_2_S treatment also significantly limited myocardial cell loss, albeit to a lower extent when compared to the slow H_2_S donor **1**. Equivalent protection was evident with RSSH donor **2** and trisulfide **4**. Intriguingly, the RSSH donor **3** showed the highest degree of post-I/R myocardial protection by limiting the total infarct to under 20% of the heart area, consistent with the functional data presented above. 

The release of cardiac troponin I (cTnI) is routinely used to determine the extent of myocyte loss after a significant ischemic event [42]. Hence, we collected the coronary effluent from I/R injured hearts at the end of reperfusion to assess the cTnI levels in I/R control and RSSH donor **3**-treated hearts. We found that total cTnI from I/R (untreated) hearts was two-fold higher than that eluted from I/R hearts receiving RSSH donor **3** at reperfusion (Figure 3C). In aggregate, these data show RSSH, given at reperfusion, effectively limits irreversible myocardial injury, thus dysfunction after global I/R, and does so in a manner that seems to outperform the protection afforded by other RSS. Based on these promising results, we chose RSSH donor **3** for further mechanistic studies.

### 3.4. RSSH Enhances H9c2 Cell Viability after Hypoxia/Reoxygenation

**Figure 4:**
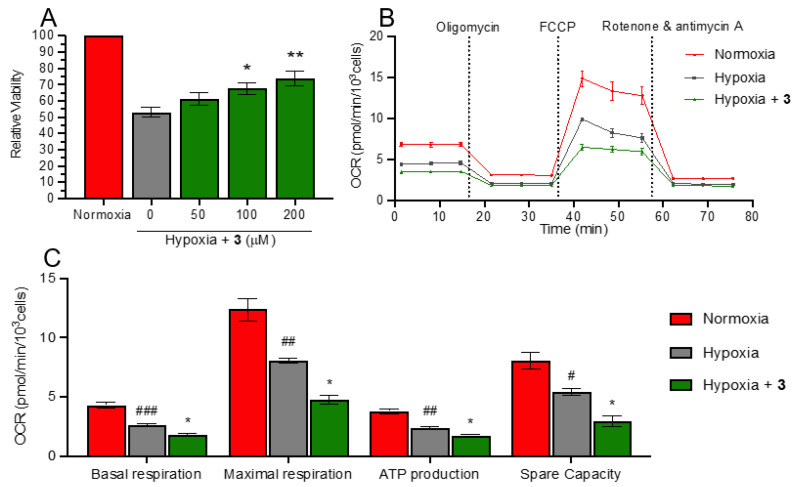
Effect of RSSH donor **3** on H9c2 cells during reoxyenation following hypoxia. (**A**) Dose-dependent protection of cell viability by compound **3** against 24 h of hypoxia followed by 3 h reoxygenation (n = 3) * *p*, 0.05, ** *p*, 0.005 vs. normoxic controls. (**B**) OCR of H9c2 cells under normoxia, or 24 h hypoxia followed by reoxygenation with or without precursor **3**. (**C**) Mitochondrial function parameters calculated from respiration data in (B) (n = 3) # *p*, 0.05, ## *p*, 0.005, ### *p*, 0.001 vs. normoxia control; * *p*, 0.05 vs. hypoxia group.

H9c2 cells are commonly used to mimic in vivo myocardial reperfusion injury, using a hypoxia/reoxygenation (H/R) approach [43,44]. Here, we used the H/R model to dissect the possible mechanisms involved in RSSH-granted protection against ischemic injury. To this end, we first placed H9c2 cells under normoxic conditions after challenging them with 24 h of hypoxia (5% CO_2_, 95% N_2_, and 1% O_2_) in substrate deficient media. This procedure resulted in an approximately 50% cell loss, as determined with the CCK-8 assay. RSSH donor **3** markedly increased H9c2 cell viability at the onset of reperfusion and dose-dependent (Figure 4A).

### 3.5. RSSH Lowers H9c2 Cell Mitochondrial Respiration Granting Cell Protection against Hypoxia-Reoxygenation

To further interrogate the mitochondrial metabolic state of H/R injured H9c2 cells, the mitochondrial respiration was evaluated by measuring OCR in Seahorse XF96 Extracellular Flux Analyzer. As shown in Figure 4B,C, the basal respiration was significantly lower in the H/R injured cells than in the normoxic group indicating that H/R specifically impaired OCR. ATP production, maximal respiration, and spare respiratory capacity were significantly lower in the H/R injured cells than in the normal group indicating that H/R decreased the energy demand due to dysfunction of mitochondria. However, RSSH donor **3** further decreased the energetic flexibility of the changes caused by H/R. When compared with the H/R viability data (Figure 4A) demonstrating the impacts of chronic ischemia on cell viability, we conclude that RSSH preserves the mitochondrial respiration in H9c2 cardiomyocytes exposed to H/R, presumably by protecting mitochondria via an induced temporary inhibition of complex IV, which is a common feature among H_2_S and related species [45]. This observation is further supported by OCR data collected from chronic (24 h) exposure to precursor **3** under normoxic conditions (Supporting Information, Figures S3 and S4). Here, we also see lowered OCR that correlate to strong viability in vitro (Supporting Information, Figure S5). These results suggest that RSSH-induced decreases in OCR under H/R or normal conditions are consistent. RSSH provides protective reduction in cellular respiration to enhance organ-protective outcomes following ischemia–reperfusion.

## 4. Discussion

H_2_S exerts many beneficial effects in the cardiovascular system [8,46,47]; however, its action mechanisms remain elusive. Emerging evidence suggests that many biological actions attributed to H_2_S may be due to H_2_S-derived RSS, including RSSH and polysulfides [14,19,48,49]. Furthermore, accumulating evidence shows RSS protection against I/R injury [24,50,51] as well as oxidative and/or electrophilic stress [35,36,52,53,54,55]. For example, Predmore and colleagues have shown diallyl trisulfide rescue from myocardial injury in a murine model of myocardial ischemia/reperfusion [20]. However, questions still arise regarding whether there are any pharmacological differences in the use of H_2_S vs. RSSH and/or polysulfides. Zhang and colleagues showed that cellular polysulfides might play a role in regulating inflammatory signaling [56]. Desensitization of macrophages to TLR4 by polysulfides negatively regulates TLR4 proinflammatory signaling which has potential implications in I/R injury. A recent study by Ke and co-workers, for instance, has shown that an H_2_S_2_ prodrug exhibits elevated analgesic effects compared to its H_2_S and RSSH analogs [57].

In the present study, we compared the biological effectiveness of distinct RSS against I/R injury. Consistent with previous reports [58], ischemic post-conditioning limits I/R injury in the isolated mouse heart. Mechanical manipulation of the flow of reperfusion allows the coronaries to modulate the coronary perfusion pressure, thereby improving endothelial cell survival and function [59]. Interestingly, IPoC has been linked to the stimulation of endogenous H_2_S production resulting in improved contractile function and limiting infarct size [10,60]. However, the effects of IPoC on aging cardiomyocytes appears to diminish requiring exogenous supplementation to achieve cardioprotection [11]. Early studies in the isolated heart have shown suppressed H_2_S production during ischemia, which was attributed to a reduction in CSE activity [61]. Cardiac-specific overexpression of CSE in mice was reported to protect against myocardial I/R injury [62]. In addition, H_2_S therapy has been shown to be effective in various ischemic diseases including models of ischemic heart disease [8,63]. Consistently, Na_2_S improved the post-ischemic functional recovery under our experimental conditions. However, inorganic sulfide salts are not ideal sources of H_2_S because of its rapid release, thus failing to mimic endogenous production and also raising toxicity concerns. Hearts treated with H_2_S donor **1** show modest improvement in the contractile rate compared with Na_2_S, suggesting the benefits of slow and sustained H_2_S release. For the RSSH donors tested here, precursor **3** (t_1/2_ = 16.7 min) shows superior cardioprotection, suggesting that the rate at which RSSH is released can significantly influence the pharmacological outcome. Given its very short lifetime, the weak beneficial actions afforded by precursor **2** (t_1/2_ = 1.4 min) compared with **3** is likely due to the rapid release of RSSH in extracellular medium, thus reduces its efficacy. In contrast, a slow RSSH release appears to be beneficial. These results suggest that not only is the type of RSS important for improved recovery post-ischemia, but the rate of delivery is also significant. Possible explanations for RSSH being a better cytoprotectant than H_2_S and RSH include stronger nucleophilic and reducing ability [54,64,65,66,67]. Furthermore, RSSH can achieve direct protein S-persulfidation on reduced thiols, whereas H_2_S-induced persulfidation is dependent on oxidized target thiol residues. We speculate that RSSH-mediated modification of protein cysteine residues protects them from irreversible modification during oxidative and/or electrophilic insult.

As previously discussed, we choose an in vitro H/R model to dissect the possible mechanism of RSSH-mediated cardioprotection. Our data reveal that RSSH indeed protect cardiomyocytes from reoxygenation injury following hypoxia, which parallels our Langendorff findings. Furthermore, RSSH preserves the mitochondrial respiration in H9c2 cardiomyocytes exposed to H/R potentially by protecting mitochondria via inhibition of oxidative phosphorylation. Importantly, our findings are consistent with previous research which shows H_2_S can reversibly induce a hypometabolic state in mice via a reduction in O_2_-consumption, CO_2_-production, and heart rate [23]. Reducing infarct size following myocardial ischemia is paramount because myocardial necrosis is a risk factor for developing heart failure [68]. Cardiac metabolism changes during ischemia, with the oxygen shortage halting oxidative phosphorylation, which depolarizes mitochondrial membranes leading to ATP depletion and overall inhibition of myocardial contractile function. When reperfusion begins, the electron transport chain is reactivated, generating ROS. The subsequent ROS is then believed to induce a variety of damage, including sarcoplasmic reticulum dysfunction and Ca^2+^ overload [69]. These abrupt metabolic changes contribute to a large extent of the reperfusion injury. Similar to the shuttering of O_2_ and nutrient in IPoC, the preserved RSSH-induced metabolic status of the myocardium in the presence of RSSH donor **3** may smooth the transition from ischemia to reperfusion by priming the cells with low O_2_ consumption, which also slows the leakage of electrons to superoxide that otherwise accumulate with high demand. A similar salutary action has been reported for the reversible complex I inhibitor, amobarbital [70,71]. Other have speculated that a H_2_S-rich environment may be utilized by invertebrates as well as by some vertebrates, as an alternative source of energy involving oxidation of H_2_S at the mitochondrial level, perhaps coupled to mitochondrial bioenergetics [72]. We note that sulfane sulfur donated by RSSH carries the same oxidation state as molecular oxygen, priming its utilization in the ETC during stress situations analogous to the previously observed effects of H_2_S [9]. Hence, reducing the rate of O_2_ consumption during early reperfusion is considered as a promising strategy to alleviate the impact of I/R injury.

The results observed with RSSH are promising, but not fully understood. We speculate that some portion of the cytoprotection might be due to the potent antioxidant capacity of RSSH, either via direct scavenging of ROS and/or activating endogenous antioxidant pathways or by RSSH-mediated modification of protein thiols to provide protection against irreversible modification. These cytoprotective features of RSSH make it an attractive candidate for therapeutic reduction in the damaging effects of hypoxia. In this study, we have demonstrated that different RSS exhibit different biological potencies, with RSSH providing the most beneficial impact.

## 5. Limitations and Future Studies

The present study comes with some limitations that deserve future, fully dedicated investigations. First, we did not assess the reversibility of RSSH-imparted modulation of mitochondrial respiratory chain function. In the current study, we observed the inhibition of oxidative phosphorylation for a duration of 24 h with RSSH precursor **3** (Supporting Information, Figures S3 and S4) that goes well beyond the half-life of this compound (16.7 min). For the time being, we can only assert that, despite their potency, RSSH impact on mitochondrial respiration is well tolerated. Another limitation is that we do not yet know whether and how decreased oxidative phosphorylation leads to in vivo cardioprotective effects. Additionally, how RSSH interact with cellular membranes remains to be determined. Previous studies have highlighted that H_2_S-related species can limit post-ischemic myocardial cell loss by limiting apoptosis/necrosis, thus preserving myocyte function [20,73]. However, these questions remain to be explored in detail specifically for RSSH-derived protection.

## 6. Conclusions

This study compared the cardioprotective effects of various RSS with Na_2_S and the canonical mechanical post-conditioning of isolated perfused mouse hearts. We observe the most positive influence on the recovery of heart function with RSSH donor **3**, whereas the other RSS species examined perform similarly to ischemic post-conditioning. All RSS were capable of reducing the amount of irreversible damage done to the heart. Further investigation of the effects of RSSH donor **3** on rodent cardiac myoblasts suggests an induction of hypometabolic status to the mitochondria of cells experiencing exposure to hypoxia and subsequent reperfusion in the presence of RSSH. The lowered metabolic demand of these cells in the early phase of reperfusion correlates with an increased viability following the hypoxic episode. We propose that by lowering the OCR and overall metabolic demand of the mitochondria, the cells avoid severe reperfusion injury. Further studies are needed to understand how exactly RSSH modulate mitochondrial metabolic/functional status.

## Figures and Tables

**Table 1:** Isolated heart parameters taken at the end of the stabilization time in the different treatment groups.

Group	n	HR (bpm)	LVDP (mmHg)	RPP ^a^ (bpm × mmHg)	+dp/dt_max_^a^ (mmHg/ms)	−dp/dt_max_^a^ (mmHg/ms)
I/R	8	370 ± 62	75 ± 16	27.8 ± 9.6	2.71 ± 0.65	−2.21 ± 0.65
IPoC	8	336 ± 63	71 ± 9	23.9 ± 8.0	2.88 ± 0.39	−2.25 ± 0.46
Na_2_S	8	349 ± 51	70 ± 12	24.4 ± 8.4	2.97 ± 0.54	−2.01 ± 0.33
**1**	8	333 ± 30	78 ± 13	26.0 ± 7.1	3.55 ± 0.87	−2.34 ± 0.50
**2**	8	385 ± 79	78 ± 18	30.0 ± 7.3	3.03 ± 1.0	−2.48 ± 0.66
**3**	8	382 ± 35	66 ± 11	25.2 ± 6.9	3.23 ± 0.46	−1.94 ± 0.33
**4**	8	323 ± 77	84 ± 23	27.1 ± 7.8	3.10 ± 0.73	−2.41 ± 0.92

^a^ Values reported as ×10^3^.

**Table 2:** Isolated heart parameters observed at the end of reperfusion in the different treatment groups.

Group	n	HR (bpm)	LVDP (mmHg)	RPP ^a^ (bpm × mmHg)	+dp/dt_max_^a^ (mmHg/ms)	−dp/dt_max_^a^ (mmHg/ms)
I/R	8	276 ± 63	25 ± 11	6.90 ± 5.3	1.16 ± 0.59	−0.61 ± 0.20
IPoC	8	256 ± 41	46 ± 13	11.8 ± 5.7	1.84 ± 0.84	−1.05 ± 0.36
Na_2_S	8	244 ± 45	36 ± 8	8.84 ± 3.8	1.45 ± 0.29	−0.79 ± 0.18
**1**	8	287 ± 45	48 ± 16	13.8 ± 7.5	2.09 ± 0.65	−1.17 ± 0.76
**2**	8	315 ± 54	45 ± 10	14.2 ± 6.1	1.55 ± 0.73	−0.96 ± 0.36
**3**	8	290 ± 47	48 ± 10	13.9 ± 5.6	2.33 ± 0.51	−1.13 ± 0.30
**4**	8	297 ± 79	46 ± 14	13.7 ± 8.9	1.41 ± 0.38	−0.93 ± 0.27

Values are mean ± SD n, number of hearts; H.R., heart rate; LVDP, left-ventricle developed pressure; +dp/dt, −dp/dt, rate of pressure rise and fall, respectively. ^a^ Values reported as ×10^3^.

## Data Availability

Data is contained within the article and Supplementary Material.

## Supplementary Materials

The following supporting information can be downloaded at: https://www.mdpi.com/article/10.3390/antiox11051010/s1. Synthesis of *N*-acetyl-*O*-ethyl cysteine trisulfide, OCR traces for H9c2 cells, and NMR spectra. Scheme S1: Synthesis of *N*-acetyl-*O*-ethyl cysteine trisulfide; Figure S1: Langendorff functional recovery at 30 min reperfusion; Figure S2: Langendorff functional recovery at 60 min reperfusion; Figure S3: OCR trace for normoxia + **3**; Figure S4: Calculated respiration parameters for normoxia + **3**; Figure S5: H9c2 viability data for normoxia + **3** vs. hypoxia + **3**.
