# Supplementary material for: Hydropersulfides (RSSH) Outperform Post-Conditioning and Other Reactive Sulfur Species in Limiting Ischemia–Reperfusion Injury in the Isolated Mouse Heart
Source: Antioxidants (Basel). 2022 May 20;11(5):1010. doi: 10.3390/antiox11051010 (PMC9137952; doi:10.3390/antiox11051010)
Supplement: Supplementary file 1 [file antioxidants-11-01010-s001.zip › antioxidants-1716807-supplementary.pdf]

Supporting Information for

Hydropersulfides (RSSH) Outperform Post-Conditioning and Other Reactive Sulfur Species in Limiting Ischemia-Reperfusion Injury in the Isolated Mouse Heart

Blaze M. Pharoah [1], Vinayak S. Khodade [1], Alexander Eremiev [1], Eric Bao [1], Ting Liu [2], Brian O'Rourke [2], Nazareno Paolocci [2,3*], and John P. Toscano [1,*]

[1]Department of Chemistry, Johns Hopkins University, Baltimore, Maryland 21218, USA

[2]Division of Cardiology, Johns Hopkins University School of Medicine, Baltimore, Maryland 21205, USA

[3]Department of Biomedical Sciences, University of Padova, Padova, Italy

[*]Correspondence: John P. Toscano, jtoscano@jhu.edu; Nazareno Paolocci, npaoloc1@jhmi.edu

Table of Contents

General information	S2
Synthesis of *N*-acetyl-*O*-ethyl cysteine trisulfide	S3
RPP, LVDP, and dP/dt_{max} % recovery at 30 and 60 minutes reperfusion	S6
Oxygen Consumption Rate (OCR) via Seahorse experiments	S7
NMR spectra of synthesized compounds	S9

General Information

Analytical thin layer chromatography (TLC) was performed on silica gel on TLC Al foils with fluorescent indicator F254 plates (Sigma-Aldrich). Visualization was accomplished with UV light (254 nm) or staining with $KMnO_4$. Starting materials, solvents, and reagents were received from commercial sources (Sigma-Aldrich, Oakwood Chemical, and TCI), unless otherwise noted and were used without purification. Deuterated solvents (Cambridge Isotope Laboratories) were used for NMR spectroscopic analyses. NMR spectra were obtained on a Bruker 400 MHz NMR spectrometer. In the case of ^1H NMR in $CDCl_3$, chemical shifts are reported relative to tetramethylsilane (δ = 0). The other spectra are referenced internally according to residual solvent signals of $CDCl_3$ (^{13}C NMR; δ = 77.16 ppm), and DMSO-d_6 (^1H NMR; δ = 2.50 ppm, ^{13}C NMR; δ = 39.52 ppm). High-resolution mass spectra were obtained on a Waters Acquity Q-ToF MS/MS instrument. The pH measurements were performed using a Fisher Scientific Accumet AB15 pH-meter.

Scheme S1. Synthesis of *N*-acetyl-*O*-ethyl cysteine trisulfide (**4**)

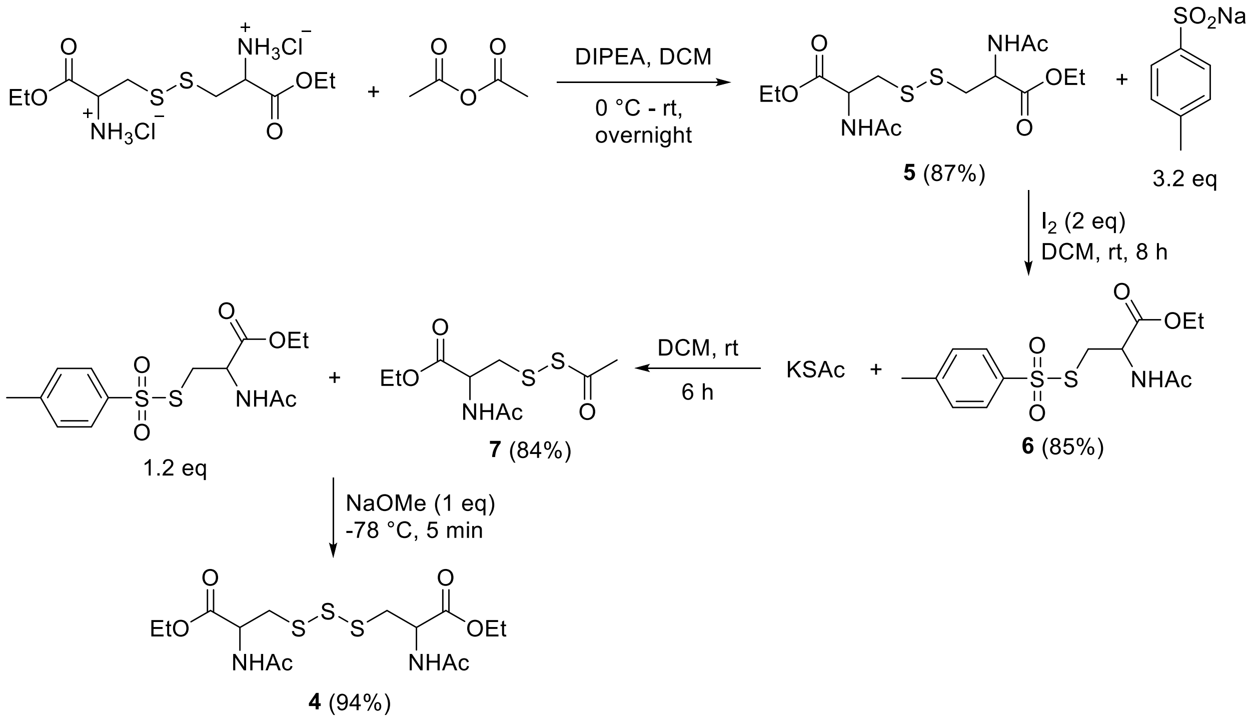

Diethyl 3,3'-disulfanediylbis(2-acetamidopropanoate) (5)

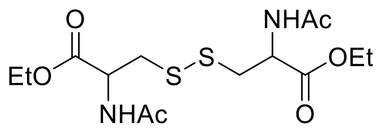

To an ice-cold suspension of *L*-cystine *bis*-ethyl ester dihydrochloride (2.0 g, 5.42 mmol) in dichloromethane (60 mL), diisopropylethylamine (3.01 g, 23.29 mmol) was added and the mixture was stirred at 0 °C for 5 min. Acetic anhydride (1.33 g, 13.00 mmol) was then added dropwise and the reaction mixture was stirred overnight at room temperature. The reaction mixture was diluted with dichloromethane (50 mL) and the organic phase was washed with water (3×50 mL), follwed by brine. The combined organic layer was dried over Na_2SO_4, filtered, and evaporated under reduced pressure. The residue was purified by silica gel flash column chromatography (hexanes/EtOAc) to yield the desired product **5** (1.8 g, 87 %) as a white solid. ^{1}H NMR (400 MHz, $CDCl_3$) δ 6.57 (d, *J* = 7.3 Hz, 2H), 4.87 (dd, *J* = 12.5, 5.1 Hz, 2H), 4.24 (q, *J* = 7.1 Hz, 4H), 3.28 – 318 (m, 4H), 2.08 (s, 6H), 1.31 (t, *J* = 7.1 Hz, 6H); ^{13}C NMR (101 MHz, $CDCl_3$) δ 170.5, 170.2, 62.1, 52.0, 40.9, 23.2, 14.2; HRMS (ESI) calc. for $C_{14}H_{24}N_2O_6S_2$ $[M+Na]^+$: 403.0968; found: 403.0974.

Ethyl N-acetyl-S-tosylcysteinate (6)

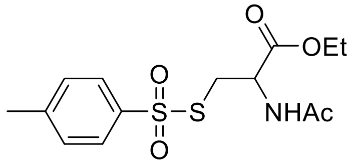

Aromatic thiosulfonate **6** was prepared according to a literature procedure.[1] Briefly, to a mixture of sodium benzenesulfinate (2.25 g, 12.62 mmol) and compound **5** (1.50 g, 3.94 mmol) in dichloromethane (40 mL), iodine (2.0 g, 7.88 mmol) was added, and the reaction mixture was stirred at room temperature for 6 h. The reaction mixture was diluted with dichloromethane (40 mL) and sodium thiosulfate (1 M) was added until the iodine color disappeared. The organic layer was separated, washed with brine, dried over Na_2SO_4, filtered, and evaporated under reduced pressure. The residue was purified by silica gel flash column chromatography (hexanes/EtOAc) to yield the aromatic thiosulfonate **6** (2.32 g, 85%) as a white solid. ^{1}H NMR (400 MHz, $CDCl_3$) δ 7.79 (d, *J* = 8.4 Hz, 2H), 7.35 (d, *J* = 8.4 Hz, 2H), 6.46 (d, *J* = 7.2 Hz, 1H), 4.84 (dt, *J* = 7.4, 4.8 Hz, 1H), 4.21 (q, *J* = 7.1 Hz, 2H), 3.47 (dd, *J* = 6.3, 4.9 Hz, 2H), 2.45 (s, 3H), 2.02 (s, 3H), 1.27 (t, *J* = 7.1 Hz, 3H); ^{13}C NMR (101 MHz, $CDCl_3$) δ 170.3, 169.6, 145.4, 141.3, 130.1, 127.3, 62.4, 51.7, 37.2, 23.0, 21.7, 14.1; HRMS (ESI) calc. for $C_{14}H_{19}NO_5S_2$ $[M+Na]^+$: 368.0579; found: 368.0602.

Ethyl N-acetyl-S-(acetylthio)cysteinate (7)

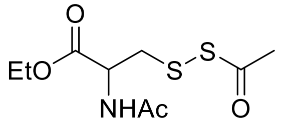

To a solution of aromatic thiosulfonate **6** (1.0 g, 2.89 mmol) in dichloromethane (30 mL), potassium thioacetate (0.429 g, 3.76 mmol) was added under a nitrogen atmosphere. The heterogeneous mixture was stirred at room temperature for 6 h. A white precipitate formed during the reaction was removed by filtration and filtrate was concentrated under reduced pressure. The crude was purified using silica gel flash chromatography (hexanes/ethyl acetate) to obtain the desired product **7** (0.65 g, 84%) as a colorless liquid. ^{1}H NMR (400 MHz, $CDCl_3$) δ 6.83 (d, *J* = 7.4 Hz, 1H), 4.79 (dt, *J* = 7.9, 4.8 Hz, 1H), 4.21 (q, *J* = 7.1 Hz, 2H), 3.40-3.07 (m, 2H), 2.43 (s, 3H), 2.07 (s, 3H), 1.29 (t, *J* = 7.1 Hz, 3H); ^{13}C NMR (101 MHz, $CDCl_3$) δ 195.1, 170.2, 170.1, 62.1, 51.3, 41.4, 29.0, 23.2, 14.2; HRMS (ESI) calc. for $C_9H_{15}NO_4S_2$ $[M+Na]^+$: 288.0335; found: 288.0341.

Diethyl 3,3'-trisulfanediylbis(2-acetamidopropanoate) (4)

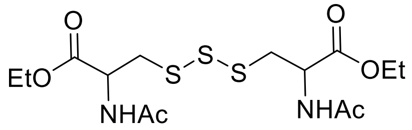

Compound **4** was prepared according to a literature procedure.[2] Briefly, *N*-acetyl-S-(acetylthio)cysteinate (0.74 g, 2.80 mmol) and aromatic thiosulfonate **6** (1.16 g, 3.36 mmol) were dissolved in THF (30 mL) under N_2 and cooled to −78 °C for 10 min. Sodium methoxide (6.17 mL, 0.5 M, 3.08 mmol) was added dropwise and the resulting solution was stirred rapidly for 5 min.

The reaction was then quenched with saturated aqueous ammonium chloride (30 mL), and the product was extracted into EtOAc (3 × 30 mL). The organic layer was washed with brine, dried over Na_2SO_4, filtered, and evaporated under reduced pressure. The residue was purified by reverse phase chromatography (acetonitrile/water) to afford the desired product **4** (1.09 g, 94%) as a white solid. ^1H NMR (400 MHz, $CDCl_3$) δ 6.57 (d, *J* = 7.5 Hz, 2H), 4.91 (dt, *J* = 7.6, 4.8 Hz, 2H), 4.29 – 4.17 (m, 4H), 3.47 – 3.38 (m, 4H), 2.06 (s, 6H), 1.30 (t, *J* = 7.1 Hz, 6H); ^{13}C NMR (101 MHz, $CDCl_3$) δ 170.3, 170.1, 62.2, 51.8, 41.5, 23.2, 14.2; HRMS (ESI) calc. for $C_{14}H_{24}N_2O_6S_3$ $[M+H]^+$: 413.0869; found: 413.0870.

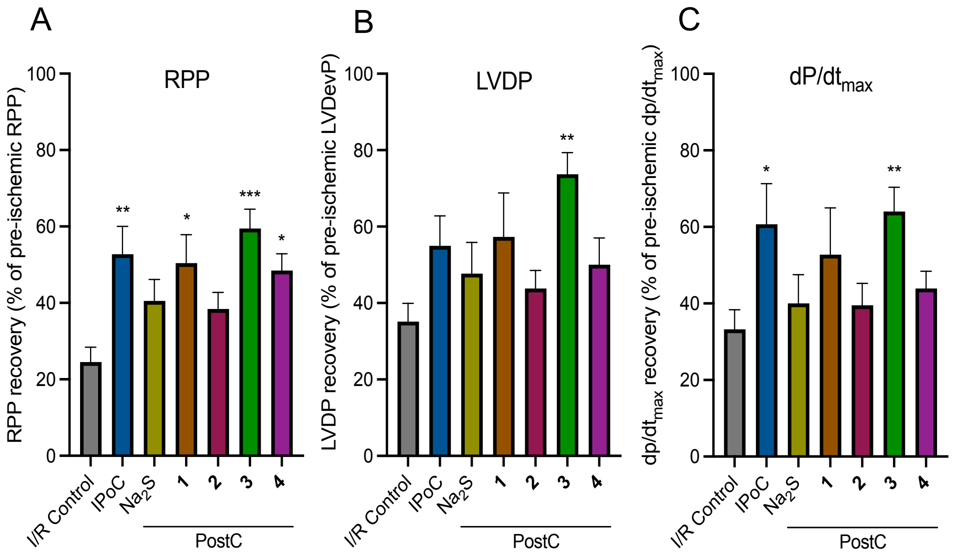

Figure S1. Cardioprotective effects induced by mechanical or pharmacological post-conditioning with different RSS at 100 μM. (A) Post-ischemic left ventricular RPP functional recovery; (B) Post-ischemic left ventricular developed pressure recovery; (C) Post-ischemic left ventricular rate of contraction. Results are expressed as the mean ± SEM using values obtained at 30 minutes of reperfusion normalized to the respective pre-ischemic value (n = 8 in each group) *P, 0.05, **P, 0.005, ***P, 0.001 vs. I/R-Control.

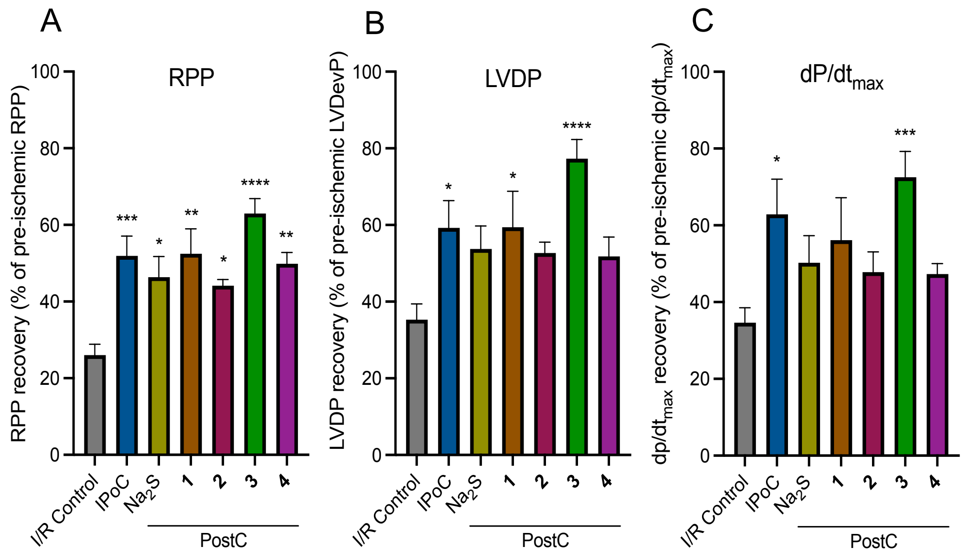

Figure S2. Cardioprotective effects induced by mechanical or pharmacological post-conditioning with different RSS at 100 μM. (A) Post-ischemic left ventricular RPP functional recovery; (B) Post-ischemic left ventricular developed pressure recovery; (C) Post-ischemic left ventricular rate of contraction. Results are expressed as the mean ± SEM using values obtained at 60 minutes of reperfusion normalized to the respective pre-ischemic value (n = 8 in each group) *P, 0.05, **P, 0.005, ***P, 0.001, **** P<0.0001 vs. I/R-Control.

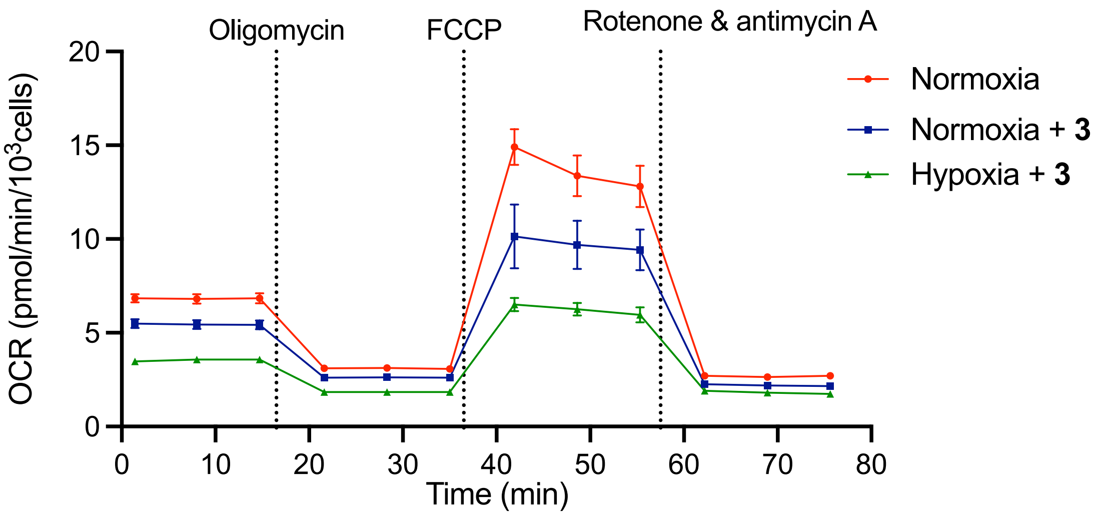

Figure S3. OCR traces for H9c2 cells undergoing 24 h incubation with RSSH precursor **3** (150 μM) under normoxia or for 3 h following 7 h hypoxia compared to control.

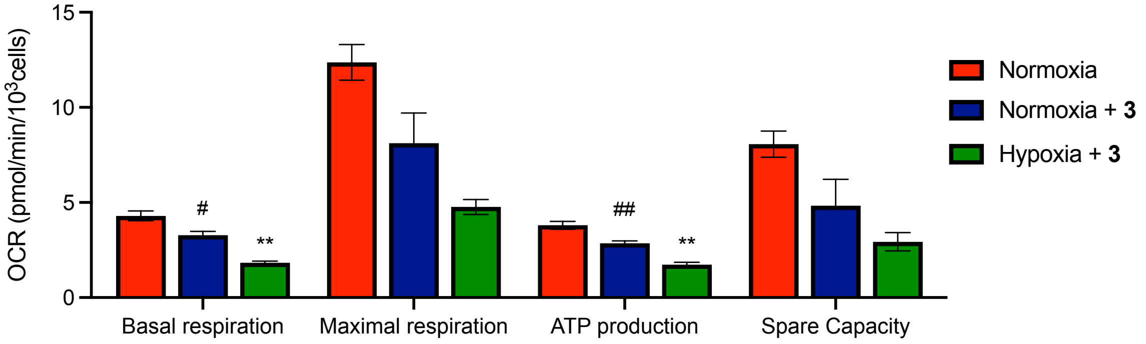

Figure S4. Calculated basal respiration, maximal respiration, ATP production, and spare capacity for H9c2 cells undergoing 24 h incubation with RSSH precursor **3** (150 μM) under normoxia or for 3h following 7 h hypoxia compared to control **(n=3)** # *p*, 0.05, ## *p*, 0.005 vs. normoxia control; ** *p*, 0.005 vs. normoxia + **3** group.

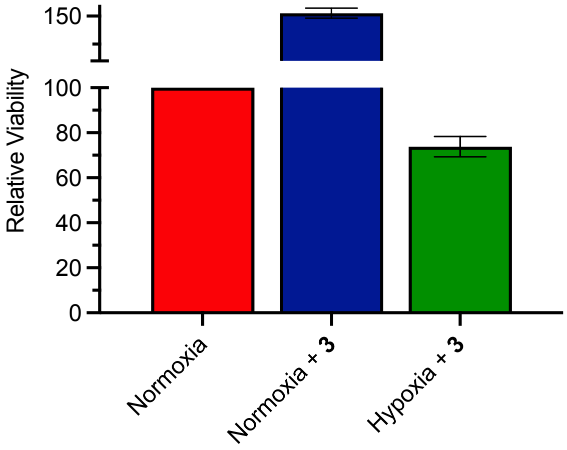

Figure S5. Comparison of cell viabilities for H9c2 cells undergoing 24 h incubation with RSSH precursor **3** under normoxia or for 3 h following 7 h hypoxia compared to control.

NMR spectra

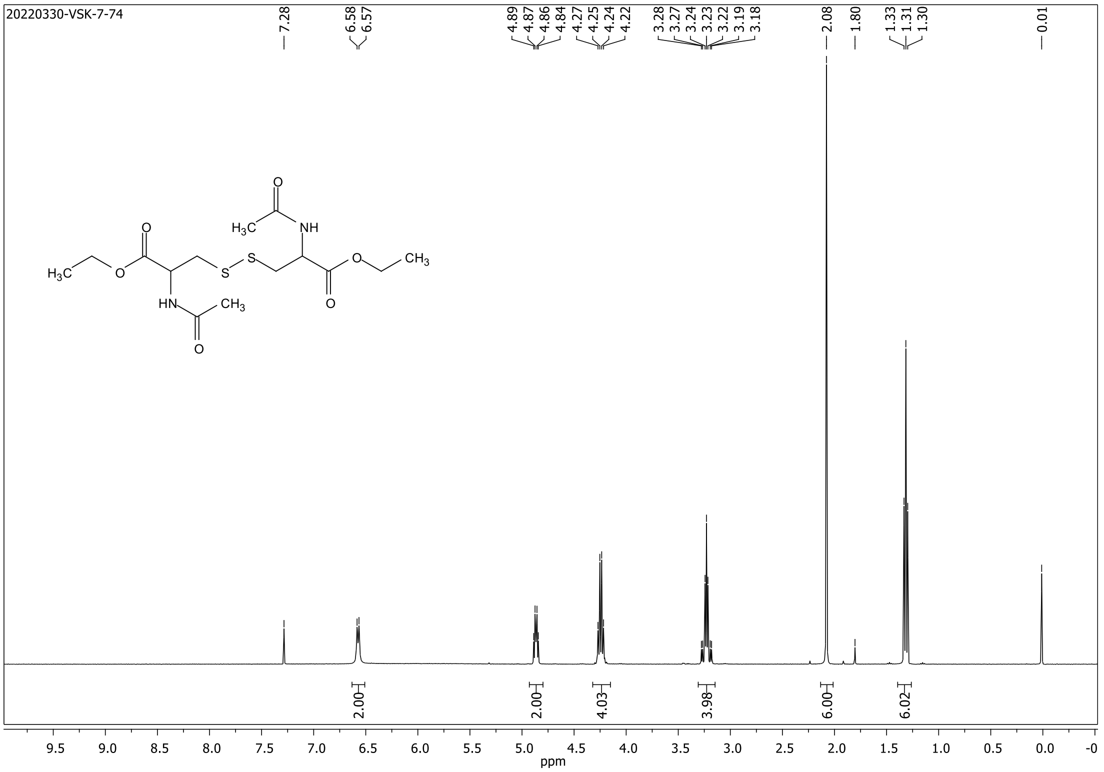

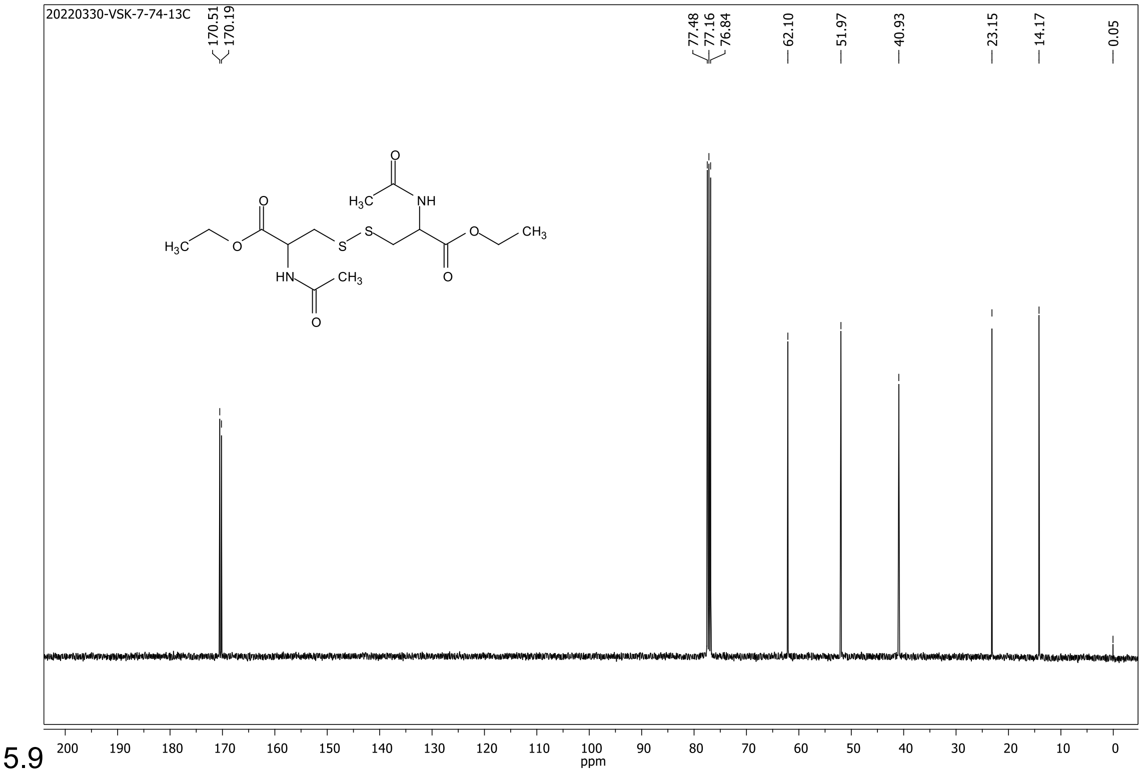

5.9

^{1}H and ^{13}C NMR spectra of compound **5**

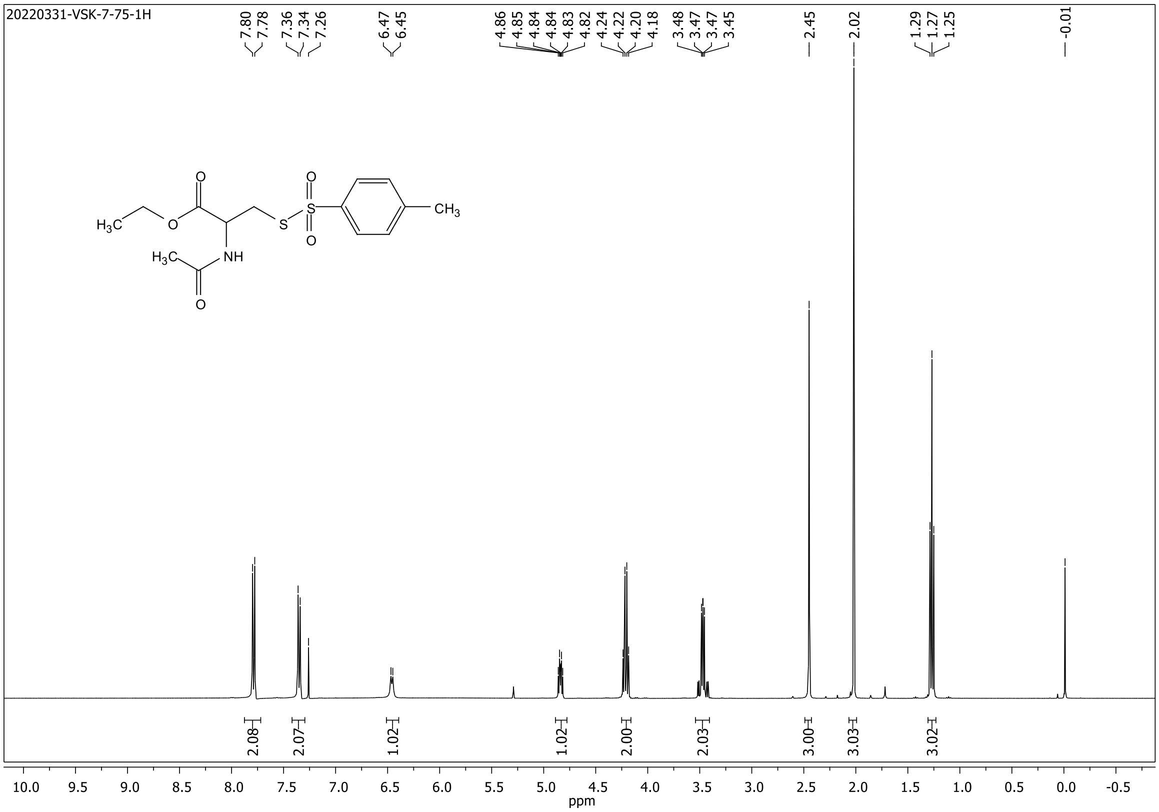

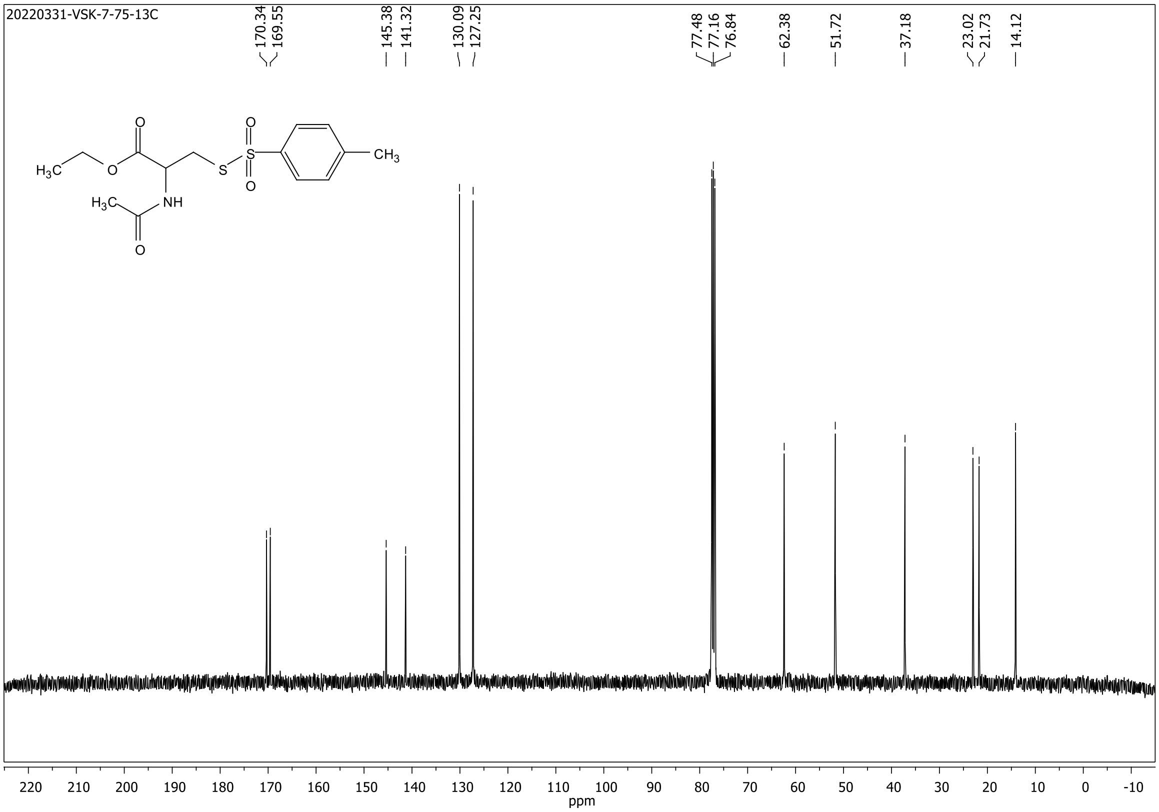

^{1}H and ^{13}C NMR spectra of compound **6**

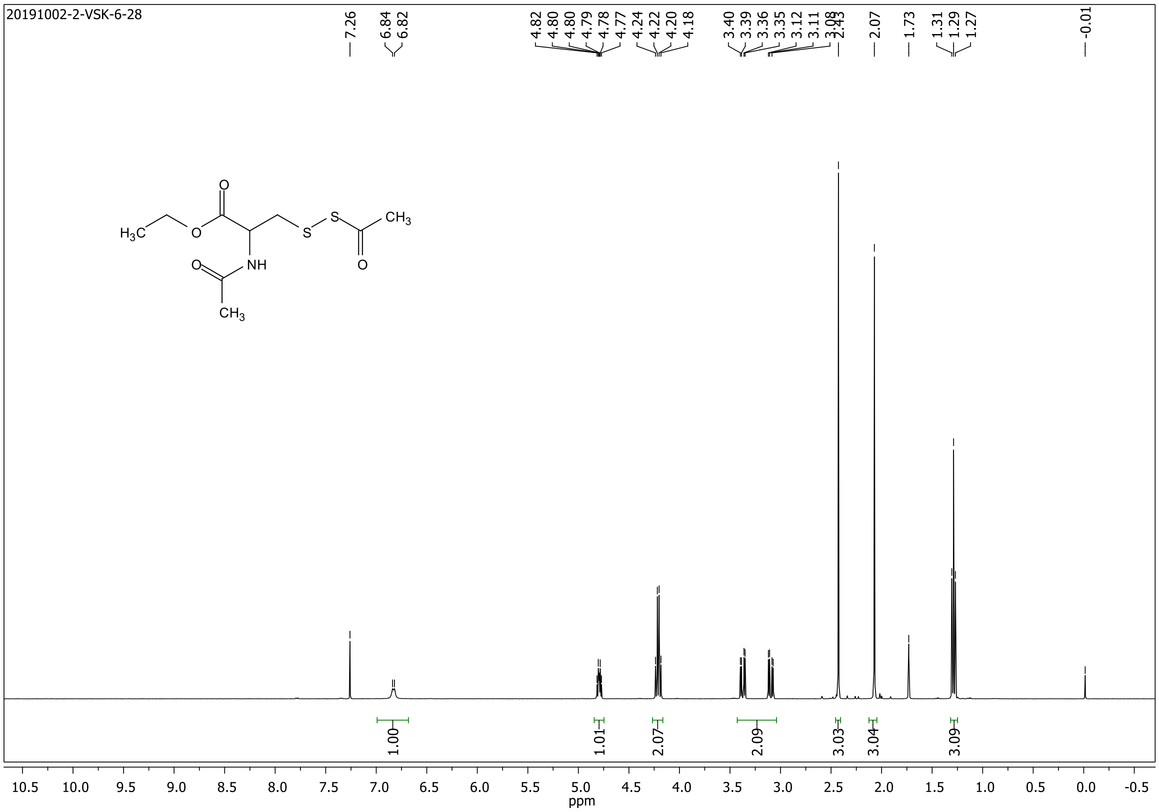

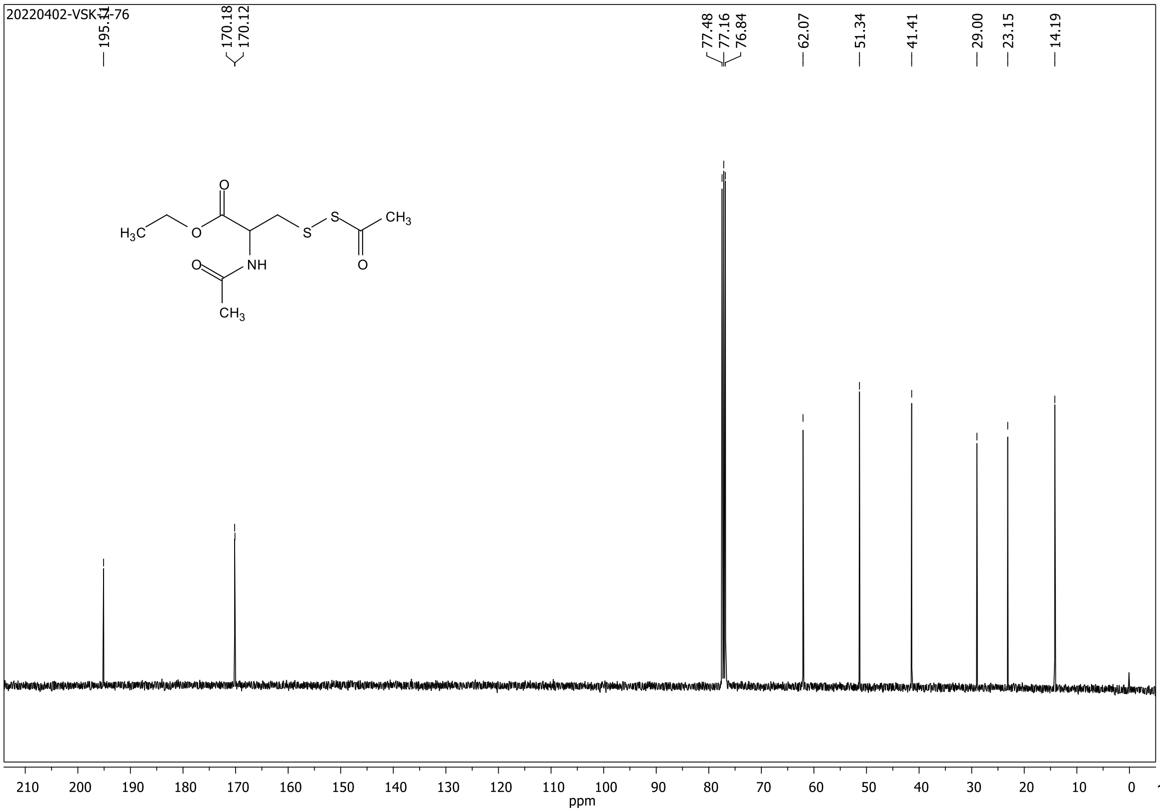

^1H and ^{13}C NMR spectra of compound **7**

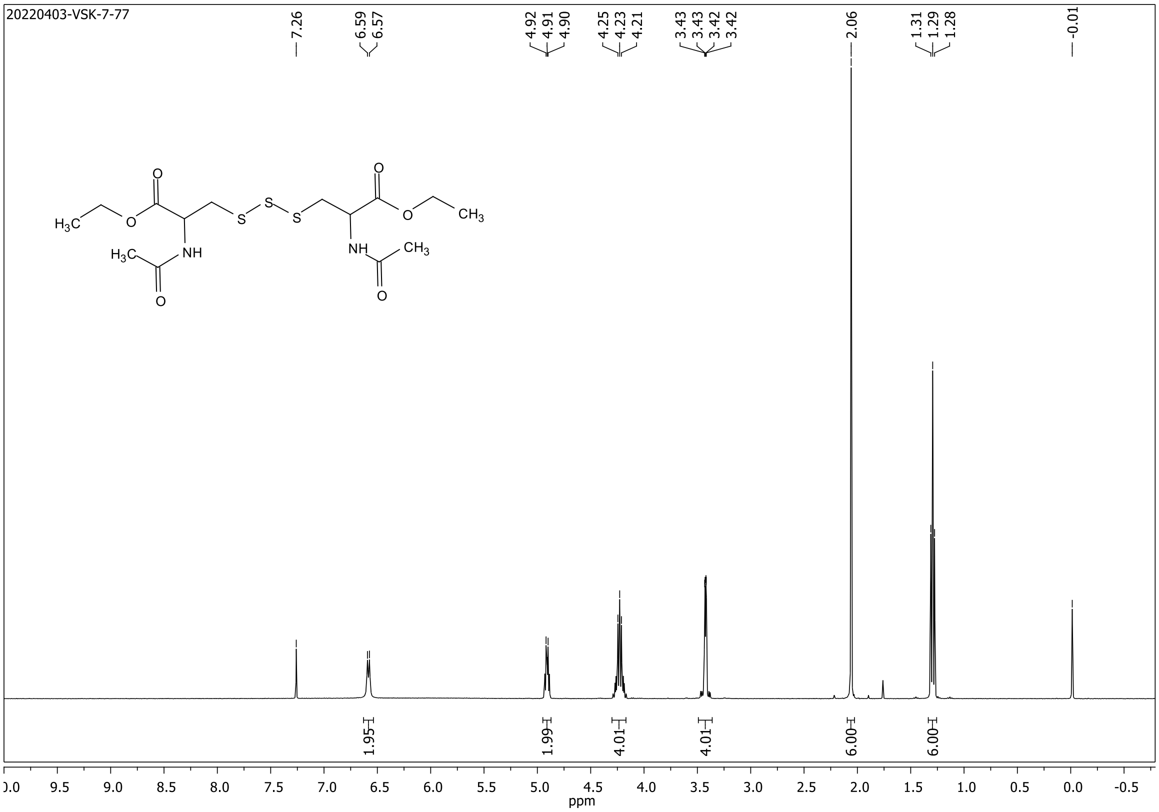

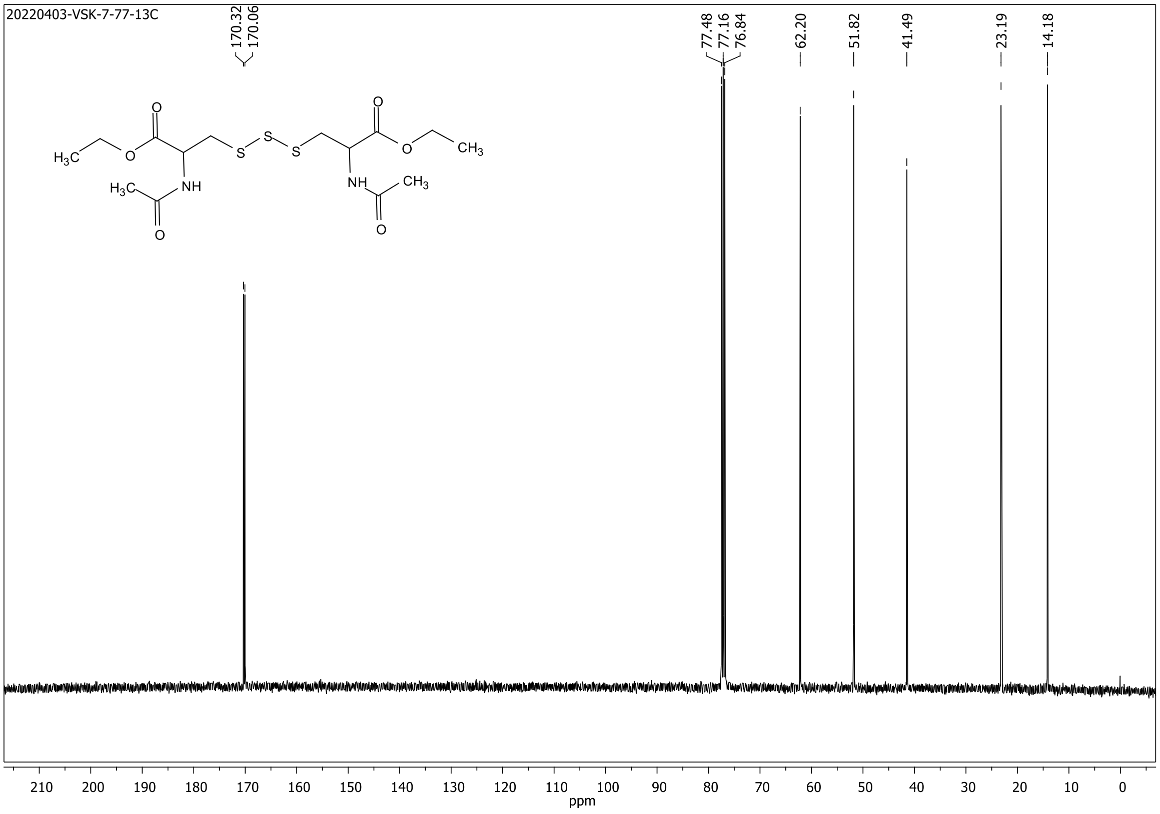

^{1}H and ^{13}C NMR spectra of compound **4**
